# Genome-Wide Identification and Analysis of the *ABCF* Gene Family in *Triticum aestivum*

**DOI:** 10.3390/ijms242216478

**Published:** 2023-11-18

**Authors:** Mila Wu, Aizhu Tu, Huimin Feng, Yunfei Guo, Gecheng Xu, Jingjing Shi, Jianping Chen, Jian Yang, Kaili Zhong

**Affiliations:** State Key Laboratory for Managing Biotic and Chemical Threats to the Quality and Safety of Agro-Products, Key Laboratory of Biotechnology in Plant Protection of Ministry of Agriculture and Rural Affairs and Zhejiang Province, Institute of Plant Virology, Ningbo University, Ningbo 315211, China

**Keywords:** wheat, *ABCF* gene family, CWMV, genome-wide, biotic stress, expression

## Abstract

The ATP-binding cassette (ABC) superfamily of proteins is a group of evolutionarily conserved proteins. The *ABCF* subfamily is involved in ribosomal synthesis, antibiotic resistance, and transcriptional regulation. However, few studies have investigated the role of *ABCF* in wheat (*Triticum aestivum*) immunity. Here, we identified 18 *TaABCF*s and classified them into four categories based on their domain characteristics. Functional similarity between *Arabidopsis* and wheat *ABCF* genes was predicted using phylogenetic analysis. A comprehensive genome-wide analysis of gene structure, protein motifs, chromosomal location, and cis-acting elements was also performed. Tissue-specific analysis and expression profiling under temperature, hormonal, and viral stresses were performed using real-time quantitative reverse transcription polymerase chain reaction after randomly selecting one gene from each group. The results revealed that all *TaABCF* genes had the highest expression at 25 °C and responded to methyl jasmonate induction. Notably, *TaABCF2* was highly expressed in all tissues except the roots, and silencing it significantly increased the accumulation of Chinese wheat mosaic virus or wheat yellow mosaic virus in wheat leaves. These results indicated that *TaABCF* may function in response to viral infection, laying the foundation for further studies on the mechanisms of this protein family in plant defence.

## 1. Introduction

The ATP-binding cassette (ABC) protein superfamily is a conserved class of proteins involved in the evolution of organisms and is widely found in bacteria, fungi, plants, and animals. ABC proteins are involved in transport, biotic stress responses, and other biochemical processes [1,2]. ABC proteins bind and hydrolyse adenosine triphosphate (ATP) to release energy for substance transport and cellular function regulation [3]. Most ABC proteins comprise two nucleotide-binding domains (NBDs) and two transmembrane domains (TMDs). NBDs are highly conserved amongst ABC proteins. However, TMDs are highly variable and transport numerous different substrates, such as hormones, pigments, toxic chemicals, secondary metabolites important for defence, lipid molecules, and reactive oxygen species (ROS)-related compounds [3]. ABC transporter proteins are numerous, and their abundance in plants is much higher than that in other organisms, probably because ABC transporter genes have undergone proliferation and diversification events during evolution, allowing plants to better adapt to the terrestrial environment [4]. Based on differences in protein size, orientation, and TMD sequences, the plant ABC transporter protein family is currently divided into eight subfamilies: ABCA–ABCG and ABCI [5]. Amongst them, all ABCA–ABCD subfamilies have a TMD–NBD organisation; the ABCG subfamily has reverse NBD–TMD organisation features; whereas soluble subfamilies E and F are more specific because they encode only two NBDs and no TMDs [6]. Previous studies have reported that ABC transporters are widely distributed in plant cell plasma membranes, mitochondria, vesicles, and peroxisomes [7]. Moreover, studies have reported that *ABCA10* plays an important role in early seed development, probably by transporting substrates for lipid metabolism into the endoplasmic reticulum [8]. Members of the ABCB gene family in *Arabidopsis* are involved in growth factor transport [9], and the overexpression of *AtABCB1* leads to hypocotyl cell elongation [10]. *AtABCC1* and *AtABCC2* mediate tolerance to both cadmium (Cd) and mercury (Hg) via vacuolar sequestration [11]. *ABCC1* is involved in transporting anthocyanidin 3-Oglucosides in grapes [12]. *ABCC4* is responsible for phytate transport [13]. *AtABCD1* is a key protein in the synthesis of benzoic acid, which is involved in interactions between plants and pathogenic bacteria [14]. *ABCG31* plays a key role in preventing water loss in plants by participating in cell-wall cuticle formation [15]. ABCG transporters are also involved in accumulating lipophilic metabolites in plant apoplasts [16]. The downregulation of *SLABCG36* and *SLABCG42* reduces cuticle deposition and thinning in tomato fruits [17].

Currently, most studies on ABC transporter proteins have focused on the ABCC and ABCG subfamilies that contain TMDs. However, few studies have been conducted on the ABCF subfamily, which comprises soluble proteins that do not act as transporters. The ABCF subfamily, also known as the general control nonderepressible (GCN) subfamily, is involved in ribosomal synthesis, antibiotic resistance, transcriptional regulation, and translational elongation. In *Arabidopsis thaliana*, this subfamily contains five members: *AtABCF1–5*. *AtABCF3* regulates stress adaptation in *Arabidopsis* by modulating the endoplasmic reticulum stress response and H_2_O_2_ uptake [18]. The N-terminal structural domain of yeast *ABCF* (also known as *GCN20*) modulates the action of ribosome-associated kinases and thus participates in regulating translation during amino acid deficiency [19]. Human *ABCF1* (also known as *ABC50*) affects translation initiation by interacting with the eukaryotic initiation factor during ribosome initiation [20]. Rice (*Oryza sativa*) *ABCF5* is also involved in stress response, and *OsABCF5* expression is significantly upregulated in roots and seedling leaves under drought and salt stress conditions. *OsABCF5* is also expressed in large quantities under Cr-rich heavy-metal stress, suggesting that *OsABCF5* is involved in the adaptation of rice to various environmental stresses [5].

Wheat is one of the most important cereal crops worldwide, and its annual production significantly affects food security. Recent studies on ABC proteins in plants have focused on the model plant *Arabidopsis thaliana*, whereas few studies have been conducted on wheat. Previous studies on ABC proteins have shown that the ABCA protein content in *Lolium perenne L.* was significantly upregulated in the presence of sodium nitrite. However, its specific function remains unclear [21]. Lr34 in wheat is a member of the plant pleiotropic drug resistance subfamily of the ABC family, which regulates responses to the leaf rust fungus *Puccinia triticina* and the wheat powdery mildew fungus *Blumeria graminis* [22]. Therefore, ABC proteins in wheat may be associated with stress responses. However, their function in viral infections has rarely been reported.

This study identified *ABCF* family members in wheat (*Triticum aestivum*) using a genome-wide analysis. Eighteen wheat ABCF genes were identified and classified into four categories based on the characteristics of their structural domains and were phylogenetically compared against their counterparts in *Arabidopsis thaliana*. Moreover, we comprehensively analysed protein motifs, protein structures, chromosome positions, and exon–intron structures. Using real-time quantitative reverse transcription polymerase chain reaction (RT-qPCR), we analysed the expression patterns of the four classes of *TaABCF* members in different tissues (roots, stems, bottom leaves, middle leaves, and top leaves) at different temperatures (8 °C, 15 °C, 20 °C, and 25 °C). We also analysed the expression patterns of four classes after treatment with different hormones, such as abscisic acid (ABA) and methyl jasmonate (MeJA), as well as the expression patterns under different viral infections, including Chinese wheat mosaic virus (CWMV), barley stripe mosaic virus (BSMV), and wheat yellow mosaic virus (WYMV). In addition, we analysed the effect of silencing *TaABCF2* on CWMV and WYMV infections using BSMV-mediated gene silencing. In conclusion, our results provide a foundation for functional studies of *TaABCF* in wheat against viral infections.

## 2. Results

### 2.1. Identification and Analysis of ABCF in Triticum aestivum

**Figure 1:**
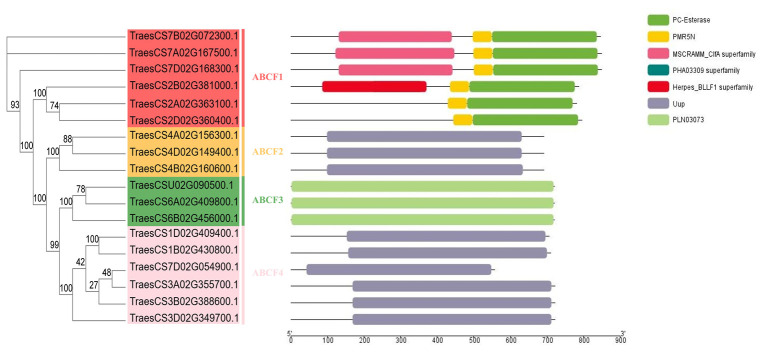
Conserved domain analysis of the TaABCF protein family. Based on the conserved domain analysis, the 18 TaABCFs could be divided into four groups: ABCF1, ABCF2, ABCF3, and ABCF4.

In this study, we conducted a whole-genome analysis to identify members of the ABCF family in the wheat genome according to the five ABCF genes in *Arabidopsis* [23]. ABCF protein sequences from *Arabidopsis* were used as query sequences for BLASTP searches of the wheat genome and full-length wheat cDNA. These results were further confirmed by a mutual BLAST analysis (Supplementary Table S1). Based on these analyses, we identified 18 genes with a similarity greater than 60% between wheat and *Arabidopsis*. For the sake of description, we classified the 18 TaABCF proteins into four classes, namely, ABCF1, ABCF2, ABCF3, and ABCF4, based on their conserved structural domains and the classification of ABCFs in *Arabidopsis thaliana*. Each class had a different conserved domain, supporting the applicability of this grouping (Figure 1). Table 1 lists the gene IDs, chromosomal loci, coding sequence (CDS) lengths, protein sizes, molecular weights, and isoelectric points. Six members of ABCF1 were between 781 and 1027 aa in length, and three members of ABCF2 were identical in amino acid length. All three members of ABCF3 had an amino acid length of 722. The six members of ABCF4 contained amino acids of a wide range of lengths, with five of them around 700 aa, and TraesCS7D02G054900.1 (558 aa) as the exception. The amino acid sequences of each category showed a high degree of similarity, and the molecular weights of the TaABCF proteins varied between 61.55 and 106.42 kDa. The isoelectric points ranged from 5.95 to 9.45. TraesCS7B02G072300.1 encoded the longest and highest-molecular-weight protein (106.42 kDa), whereas TraesCS7D02G054900.1 encoded the shortest and lowest-molecular-weight protein (61.55 kDa) (Table 1). The protein characteristics of TaABCFs were similar to those of ABCFs from other plant species [5,24].

### 2.2. Phylogenetic Analysis of the ABCF Proteins

**Figure 2:**
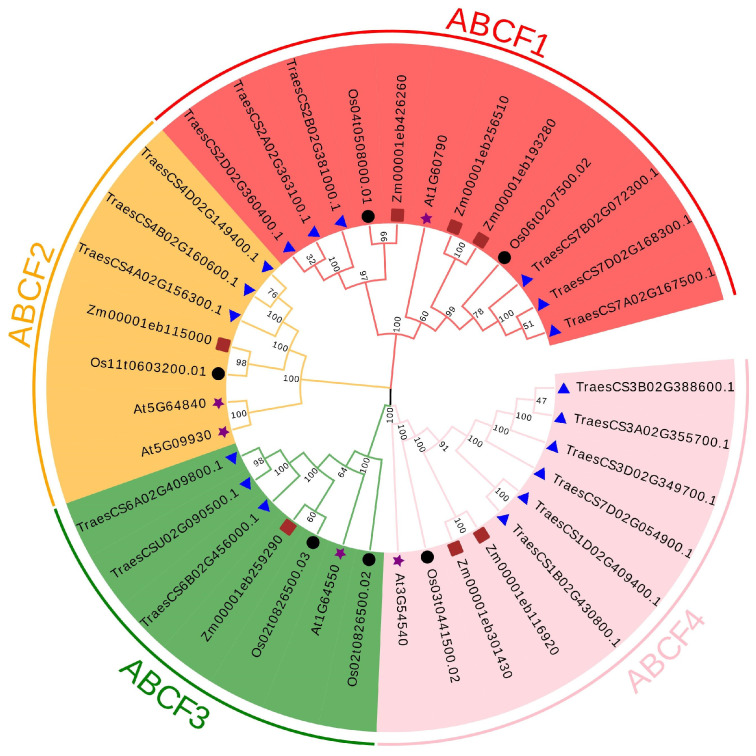
A phylogenetic tree of ABCF proteins in *Arabidopsis*, wheat, rice, and maize constructed using the neighbour-joining method in MEGA-X. The number at the node represents the guidance value after 1000 iterations. Each group is represented by a different colour. Stars represent *Arabidopsis*, triangles represent wheat, circles represent rice, and rectangles represent maize.

To analyse the phylogenetic relationships amongst the ABCFs of different species, an adjacent junction tree was constructed using MEGA-X with 5 *Arabidopsis* (diploid), 18 wheat (hexaploid), 6 rice (diploid), and 7 maize (*Zea mays*) (diploid) ABCF protein sequences. TaABCFs, OsABCFs, and ZmABCFs were highly homologous to *Arabidopsis* ABCFs. As expected, ABCF proteins from the four species were divided into four branches, indicating that these subfamilies were present in both dicotyledons and monocotyledons. The ABCF1 and ABCF4 subfamilies had six members each, whereas the ABCF2 and ABCF3 subfamilies had three members each (Figure 2).

### 2.3. Tertiary Structure Models of ABCF Protein

**Figure 3:**
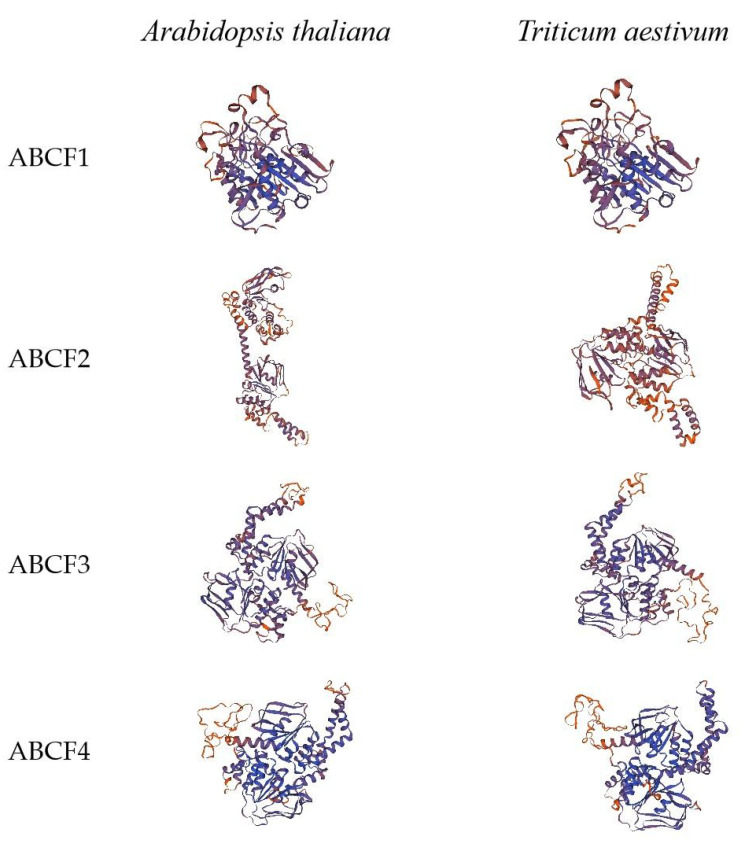
Predictive structure of ABCF proteins in *Arabidopsis* and wheat. Randomly selected protein models from each group: ABCF1 (*AT1G60790*, *TraesCS7B02G072300.1*); ABCF2 (*AT5G64840*, *TraesCS4A02G156300.1*); ABCF3 (*AT1G64550*, *TraesCS6A02G409800.1*); and ABCF4 (*AT3G54540*, *TraesCS3B02G388600.1*). SWISS-MODEL was used for structural prediction. Based on QMEAN and GMQE, the model with the optimum results was selected.

Homology modelling is an important technique in structural biology [25]. To gain insight into the structural characteristics of the TaABCF proteins, we randomly selected one protein from each group of *Arabidopsis* and wheat. Then, we generated a three-dimensional protein model using SWISS-MODEL. The results showed that ABCF1, ABCF3, and ABCF4 proteins had similar structures in different species, whereas the ABCF2 protein structure appeared to vary from species to species (Figure 3). Collectively, the proteins from different species within the same subfamily exhibited similar structures, whereas those from different subfamilies within the same species exhibited considerable differences. This result revealed the structural diversity of the ABCF family in the two species.

### 2.4. Analysis of TaABCF Structure and Conserved Motifs

**Figure 4:**
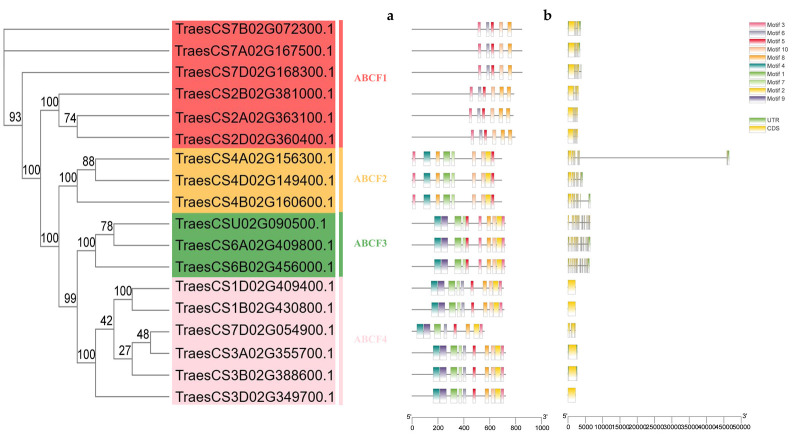
Gene structures and conserved motifs of *TaABCF*. (**a**) Distribution of all motifs identified by MEME. Differently coloured frames represent different protein motifs, and each motif has its own number. (**b**) Exon–intron structures of 18 *TaABCF* genes. Exons, introns, and untranslated regions are indicated by yellow frames, grey lines, and green frames on the right, respectively.

To characterise the putative motifs in the wheat *ABCF* family, we submitted the predicted amino acid sequences of the 18 TaABCF proteins to the MEME website and plotted the results using TBtools (Version: 1.098768). Ten conserved motifs were identified in the proteins. The distribution of these motifs in TaABCF is shown in Figure 4a. The motif distribution patterns of TaABCF proteins in the same subfamily were similar, indicating that these proteins may have similar functions [26]. Motifs 3 and 5 were present in each subfamily, with the *ABCF3* subfamily having the highest number of motifs. To gain insight into the evolution of gene families, we analysed the exon–intron structure of *TaABCF* [27]. Genomic DNA sequence analysis revealed that the number of exons varied from 1 to 16. Genes in the same subfamily had similar exon numbers; however, their exon and intron lengths differed. Amongst all subfamilies, *TaABCF3* had the highest number of exons (Figure 4b).

### 2.5. Chromosomal Location, Synteny Analysis, and Duplication Events of TaABCF

**Figure 5:**
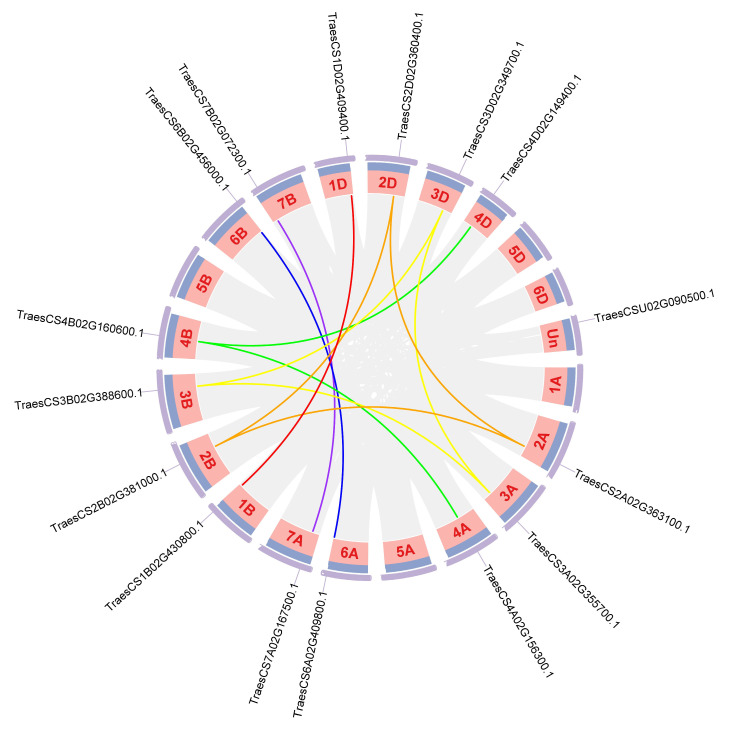
Synteny analysis and chromosomal localisation of *ABCF*s in wheat. Differently coloured lines indicate duplicated *TaABCF* pairs on different chromosomes: red lines indicate duplicated *TaABCF* pairs on chromosomes 1, orange lines indicate duplicated *TaABCF* pairs on chromosomes 2, yellow lines indicate duplicated *TaABCF* pairs on chromosomes 3, green lines indicate duplicated *TaABCF* pairs on chromosomes 4, blue lines indicate duplicated *TaABCF* pairs on chromosomes 6, purple lines indicate duplicated *TaABCF* pairs on chromosomes 7. Grey lines indicate synthesis results for the *T. aestitum* genome, and the position of *TaABCF* is marked directly on the chromosome.

Because wheat is a hexaploid plant containing three subgenomes (A, B, and D), each wheat gene can have orthologues on three homologous chromosomes [28]. Amongst the 18 predicted *TaABCF* family genes, three *TaABCF* genes were located on chromosomes 2, 3, and 4; two *TaABCF* genes were located on chromosomes 1 and 6; four *TaABCF* genes were located on chromosome 7; one *TaABCF* gene was located on chromosome Un; and none were found on chromosome 5. The three *TaABCF* members on chromosomes 2, 3, and 4 were evenly distributed in subgenomes A, B, and D, respectively, whereas the two *TaABCF* members on chromosomes 1 and 6 were distributed on chromosomes 1 B and 1 D, and 6 A and 6 B, respectively (Supplementary Figure S1). To understand this situation in hexaploid wheat and determine the chromosomal loci and replication relationships of all *TaABCF* genes, we evaluated the tandem replication events in the *TaABCF* family using Circos. The results showed that amongst the 18 *TaABCF* members, 11 segmental duplication pairs were identified (Figure 5). In genetics, Ka/Ks represents the ratio between the non-synonymous substitution rate (Ka) and synonymous substitution rate (Ks) of two protein-coding genes. This ratio determines whether selection pressure acts on protein-coding genes [29]. Chromosomal covariance and homozygosity analyses identified 11 putative paralogues (Ta–Ta) in wheat. The Ka/Ks ratios ranged from 0.0327 to 0.5566, and all duplicated *TaABCF* gene pairs had Ka/Ks ratios lower than 1. *ABCF* pairs were considered for purifying selection. Based on the differentiation rate of 9.1 × 10^−9^ synonymous mutations per year per homozygous locus, the equation *T* = Ks/(2 × 9.1 × 10^−9^) Mya was used to assess the differentiation time (T). It was determined that the 11 homozygous (Ta–Ta) member pairs diverged between 2.007 and 6.107 Mya (Table 2).

### 2.6. Prediction and Analysis of Cis-Acting Elements in the Promoter Regions of TaABCF Genes

**Figure 6:**
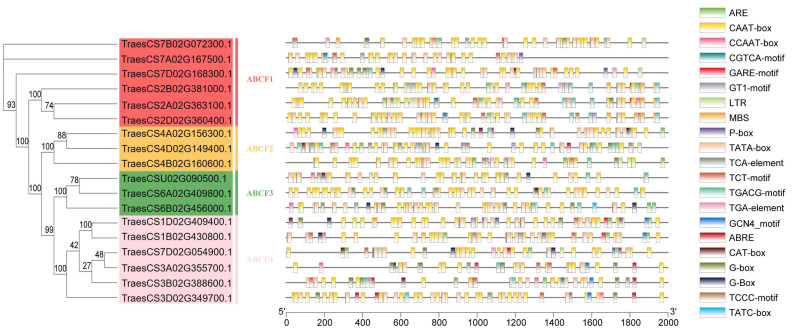
Prediction of cis-acting elements of *TaABCF*. *TaABCF* genes are shown on the left. Distinct colours indicate different subfamilies, and differently coloured boxes indicate different cis-acting elements. Names of cis-acting elements are shown on the right.

The regulation of gene expression at the promoter level is primarily controlled by cis-acting elements located upstream of the transcription start site [30]. To understand the role of cis-regulatory elements of *TaABCF*s in biotic and abiotic stress-specific responses, we analysed the promoter regions of the 18 *TaABCF* members using the PlantCARE database and predicted 2526 cis-acting elements. These elements were associated with oxygen, hormone response, light response, promoter and enhancer elements, binding-site elements, and development. The most abundant elements were those involved in responses to hormones such as ABA; MeJA; gibberellin acid (GA); salicylic acid (SA); and indole-3-acetic acid (growth hormone, IAA), indicating that the *TaABCF* gene family was significantly influenced by hormonal factors. Most *TaABCF*s encoded elements associated with environmental stress, including those essential for anaerobic induction, low-temperature-responsive elements, and drought-responsive elements. A subset of *TaABCF*s was found to encode developmentally relevant elements, including cis-regulatory elements determining *TaABCF* expression associated with meristematic and endosperm tissues (Figure 6).

### 2.7. Tissue-Specific Analysis of TaABCF Expression

**Figure 7:**
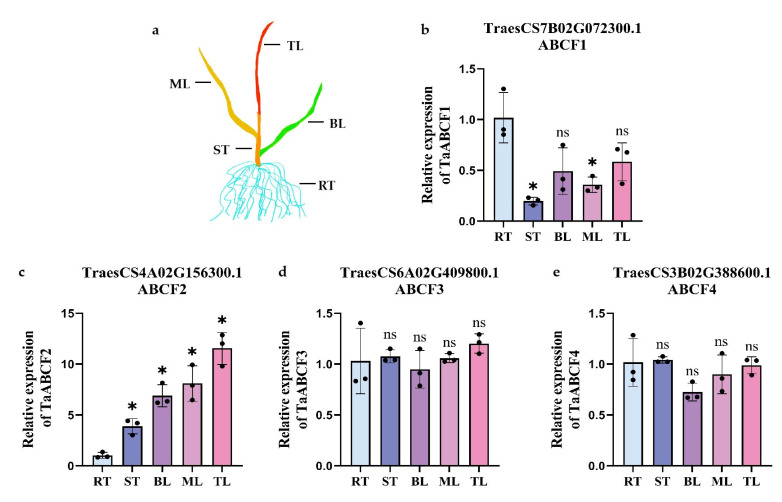
Tissue-specific differential expression of *TaABCF*s. (**a**) Model for five tissue types of wheat. TL, top leaf; ML, middle leaf; BL, bottom leaf; ST, stem; RT, root. (**b**–**e**) Relative expression of *TaABCF*s. Three independent biological replicates were used to calculate the mean expression of *TaABCF*s in other tissues relative to that in roots. The asterisks indicate significant differences determined by Student’s *t*-test (* *p* < 0.05). ns, no significant difference. Error bars, results are shown as mean ± SD.

To investigate the expression patterns of TaABCF genes, one *TaABCF* from each subfamily was randomly selected and analysed for expression in three-leaf seedlings using RT-qPCR. Plants were categorised into five tissue types: top leaves, middle leaves, bottom leaves, stems, and roots. The results showed that the four *TaABCF* members were expressed at different levels in different tissues (Figure 7). Amongst them, *TaABCF1* was expressed at low levels in all tissues except roots, whereas *TaABCF3* and *TaABCF4* showed moderate expression in all plant tissues. Notably, *TaABCF2* showed high expression in tissues other than the roots, with the highest expression in wheat leaves, followed by the stems and roots. These results suggested that the expression pattern of *TaABCF* varies according to the tissue type and is associated with plant development.

### 2.8. Expression Patterns of TaABCFs under Different Stresses

**Figure 8:**
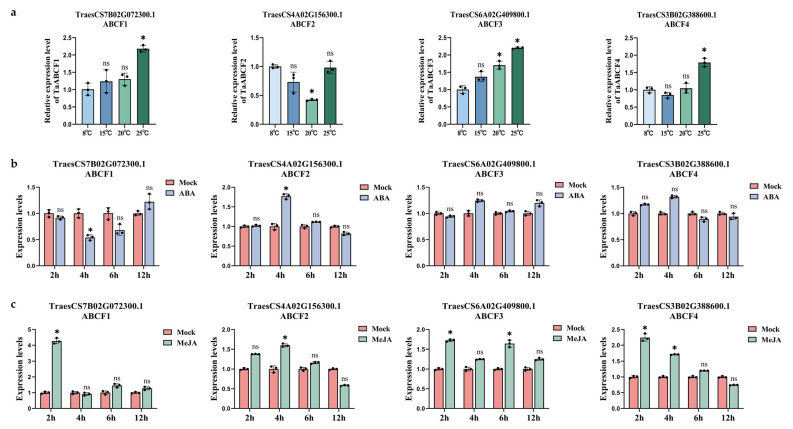
Expression of *TaABCF*s under different stresses. (**a**) Relative expression of *TaABCF* was measured using RT-qPCR in plants grown at different temperatures for 14 d. Mean expression values were calculated from three independent biological replicates and three technical replicates. The 8 °C treatment was used as a control. (**b**,**c**) Relative expression of four *TaABCF* genes in the leaves of wheat seedlings at 2, 4, 6, and 8 h after ABA or MeJA hormone treatment. Treatment after 2 h was used as a control. Three biological replication experiments were performed for each treatment, and gene expression was detected via RT-qPCR and visualised using GraphPad Prism 9.5 software. The asterisks indicate significant differences determined using Student’s *t*-test (* *p* < 0.05). ns, no significant difference. Error bars, results are shown as mean ± SD.

To investigate the effects of biotic and abiotic stressors on *TaABCF* expression, we examined the relative expression of *ABCF*s in wheat at different temperatures. The expression patterns of the four TaABCF genes in the second leaf of 14-day-old wheat were determined using RT-qPCR (Figure 8a). The results showed that *TaABCF*s exhibited lower expression at lower temperatures. *TaABCF3* showed significantly higher expression at 25 °C compared to 8 °C. *TaABCF1* and *TaABCF4* showed no significant change in expression under low and intermediate temperatures (8 °C, 15 °C, and 20 °C). However, the expression of *TaABCF2* was significantly lower at 20 °C than at 8 °C. All genes showed higher expression at higher temperatures (25 °C).

We investigated the roles of TaABCF genes in hormone responses because phytohormones play an important regulatory role in plant growth and development, as well as regulating plant responses to both abiotic and biotic stresses [31]. One TaABCF gene with hormone regulatory sites (for ABA and MeJA) was selected from each group, and the effect of hormonal stress on *TaABCF* was verified. The results showed that *TaABCF1* and *TaABCF2* were highly sensitive to both ABA and MeJA. *TaABCF3* and *TaABCF4* were less sensitive to ABA, but very sensitive to MeJA (Figure 8b,c). This result confirmed the existence of hormone-responsive cis-acting elements.

### 2.9. Analysis of TaABCF Expression after Different Viral Infections

**Figure 9:**
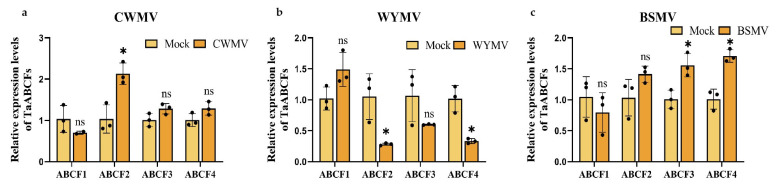
Relative expression of *TaABCF* was detected by RT-qPCR in plants inoculated with different viruses. (**a**) Changes in gene expression of wheat seedlings 14 d after CWMV infection. Healthy wheat samples from the same lot were used as controls. (**b**) Changes in gene expression of wheat seedlings after 14 d of WYMV infection. Healthy wheat samples from the same lot were used as controls. (**c**) Detection of changes in gene expression in wheat seedlings after 14 d of BSMV infection; plants inoculated with 1 × FES buffer (Mock) were used as a negative control. All expression values are presented as mean ± SEM and were calculated using three independent biological replicates and three technical replicates. The asterisks indicate significant differences determined by Student’s *t*-test (* *p* < 0.05). ns, no significant difference. Abbreviations: BSMV, barley stripe mosaic virus; CWMV, Chinese wheat mosaic virus; RT-qPCR, real-time quantitative reverse transcription polymerase chain reaction; SEM, standard error of the mean; WYMV, wheat yellow mosaic virus.

To investigate the relationship between the *TaABCF* family and plant viruses, we examined the expression of *TaABCF* in wheat inoculated with BSMV, CWMV, or WYMV. Fourteen days after inoculation, all the plants were successfully infected with BSMV, CWMV, or WYMV (Supplemental Figure S2). The results showed that the expression of *TaABCF* exhibited different changes following infection with different viruses. The expression of *TaABCF2* increased 14 d after CWMV infection, as determined using RT-qPCR (Figure 9a). Notably, the relative expression of *TaABCF2* was significantly reduced at 14 d after WYMV infection (Figure 9b). Additionally, after the same period of BSMV infection, we observed the increased expression of *TaABCF3* and *TaABCF4* (Figure 9c). Healthy wheat samples from the same lot were used as controls. These results suggest that *TaABCF2* is considerably influenced by CWMV and WYMV and may play a role in the post-infection response. Therefore, we conducted further experiments on *TaABCF2* to investigate its mechanism of action in CWMV and WYMV infections.

### 2.10. Functional Analysis of TaABCF2 in Wheat Resistance to CWMV or WYMV Infection

**Figure 10:**
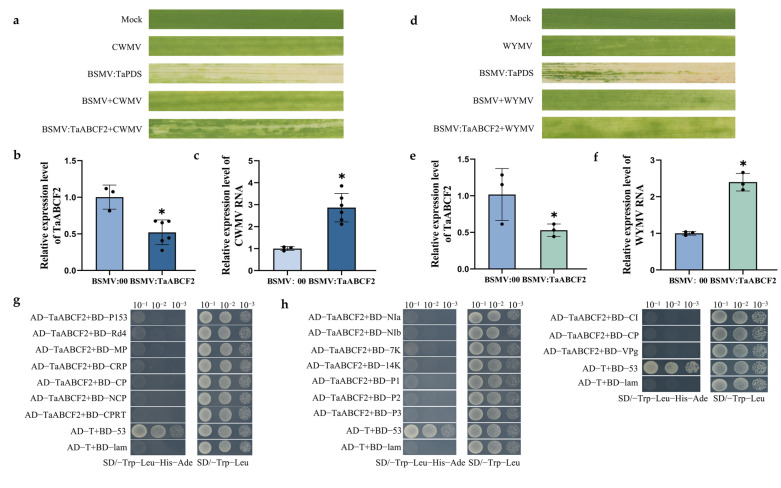
TaABCF2 was involved in wheat resistance to CWMV or WYMV infection. (**a**) Mosaic symptoms on wheat leaves infected with CWMV; BSMV:TaPDS (phytoene desaturase gene, acting as a positive control); BSMV:00 + CWMV; or BSMV:TaABCF2 + CWMV. Mock leaves were inoculated with excess inoculation buffer as a control. Photographs were taken at 21 dpi. (**b**) Relative expression of *TaABCF2* in CWMV-inoculated wheat plants was analysed using RT-qPCR. (**c**) The accumulation of CWMV RNA during *TaABCF2* silencing was analysed via RT-qPCR at 21 dpi using CWMV CP-specific primers. (**d**) Mosaic symptoms in the wheat leaves infected with WYMV, BSMV:TaPDS, BSMV:00 + WYMV, or BSMV:TaABCF2 + WYMV. (**e**) Relative expression of *TaABCF2* in WYMV-inoculated wheat plants was analysed using RT-qPCR. (**f**) The accumulation of WYMV RNA during *TaABCF2* silencing was analysed using RT-qPCR at 21 dpi using WYMV CP-specific primers. (**g**) Yeast two-hybrid (Y2H) assay to verify the interaction of *TaABCF2* with CWMV-related proteins. AD-TaABCF2 was co-expressed with BD-CRP, BD-CP, BD-P153, BD-Rd4, BD-MP, BD-NCP, and BD-CPRT in yeast cells, and the transformed cells were grown on SD/−Leu/−Trp medium and then on SD/−Trp/−Leu/−His/−Ade medium to determine protein–protein interactions. Yeast cells co-transformed with AD-T + BD-lam were used as a negative control, and yeast cells co-transformed with AD-T + BD-53 were used as a positive control. (**h**) Y2H assay to verify the interaction of *TaABCF2* with WYMV-associated proteins. AD-TaABCF2 was co-expressed with BD-NIa, BD-NIb, BD-7K, BD-14K, BD-P1, BD-P2, BD-P3, BD-CI, BD-CP, and BD-VPg in yeast cells, and the transformed cells were grown on SD/−Leu/−Trp medium and then on SD/−Trp/−Leu/−His/−Ade medium to determine protein–protein interactions. Yeast cells co-transformed with AD-T + BD-lam were used as a negative control, and yeast cells co-transformed with AD-T + BD-53 were used as a positive control. All RT-qPCR data are presented as means ± SD, as determined using the Student’s *t*-test. Each treatment had three biological replicates (* *p* < 0.05). Abbreviations: BSMV, barley stripe mosaic virus; CWMV, Chinese wheat mosaic virus; RT-qPCR, real-time quantitative reverse transcription polymerase chain reaction; SD, standard deviation; Y2H, yeast two-hybrid; WYMV, wheat yellow mosaic virus.

RT-qPCR analysis showed that *TaABCF2* expression was considerably upregulated after CWMV infection but downregulated after WYMV infection (Figure 9a,b). To investigate the role of *TaABCF*s in CWMV and WYMV infections, we silenced *TaABCF2* expression using a BSMV-induced gene silencing assay (BSMV-VIGS). Two-leaf-stage wheat seedlings were inoculated with BSMV:TaABCF2, BSMV:00, or BSMV:TaPDS (the phytoene desaturase gene acted as a positive control and exhibited a typical photobleaching phenotype), where the BSMV:00 and BSMV:TaPDS inoculated plants were used as controls. Seven days post-inoculation (dpi), wheat seedlings were co-inoculated with CWMV, BSMV + CWMV, or BSMV:TaABCF2 + CWMV. After 14 dpi, RT-qPCR confirmed that all co-inoculated plants were successfully infected with BSMV and CWMV (Supplemental Figure S3). The mosaic symptoms were significantly stronger in the BSMV:TaABCF2 + CWMV co-inoculated plants than in the BSMV + CWMV co-inoculated plants (Figure 10a). Additionally, the RT-qPCR analysis of plants co-inoculated with BSMV:TaABCF2 + CWMV using specific primers (Supplemental Table S2) confirmed that *TaABCF2* expression was reduced by approximately 50% (Figure 10b). We found that the accumulation of CWMV CP (capsid protein) in the BSMV:TaABCF2 + CWMV co-inoculated plants was much higher than that in the BSMV + CWMV co-inoculated plants (Figure 10c). In addition, after silencing *TaABCF2* by approximately 50% and inoculating with WYMV (Figure 10e), the accumulation level of WYMV CP in the BSMV:TaABCF2 + WYMV co-inoculated plants was much higher than that in the BSMV + WYMV co-inoculated plants (Figure 10f). The phenotypes of wheat leaves were consistent with our RT-qPCR results (Figure 10d). All the plants were successfully infected with BSMV and WYMV (Supplemental Figure S4). These results indicated that *TaABCF2* plays an important role in wheat resistance to CWMV and WYMV.

### 2.11. A Model Summarising the Function of TaABCFs in the Response of Wheat to Virus Infection

**Figure 11:**
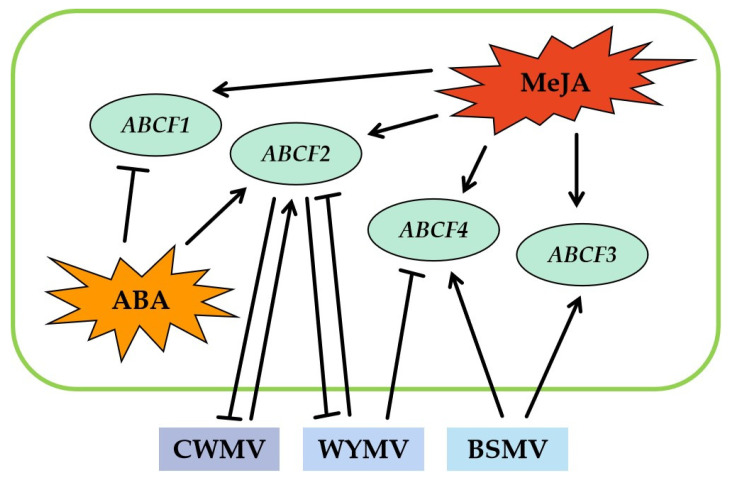
A model of the function of *TaABCF*s during virus infection. *TaABCF2* in wheat can promote infection by CWMV and WYMV. The arrows represent positive regulation, perpendicular lines represent negative regulation, and solid lines represent results by determination.

Based on the results of this study, we developed a model showing the function of *TaABCF*s in wheat during viral infection (Figure 11). RT-qPCR experiments revealed that MeJA treatment increased the expression of the four *TaABCF*s. Following ABA treatment, the expression of *TaABCF1* decreased, whereas that of *TaABCF2* increased. However, ABA had no significant effect on the expression of *TaABCF3* or *TaABCF4*. Only *TaABCF2* was upregulated in expression. Both *TaABCF2* and *TaABCF4* were downregulated after WYMV infection, whereas the other two genes showed no significant changes. After BSMV infection, *TaABCF3* and *TaABCF4* were upregulated. Using BSMV-mediated gene silencing experiments, we demonstrated that *TaABCF2* increased the occurrence of CWMV and WYMV infections.

## 3. Discussion

ABCF proteins do not exist in archaea but are widespread in eukaryotes and prokaryotes. Most organisms encode multiple ABCF family members. Previous studies have reported that all members of the ABCF family are translation factors that can bind to the E site of tRNA on the ribosome and participate in regulating the conformation of the peptidyl transferase centre conformation [32,33,34,35]. The identification of the ABCF gene family at the genomic level using bioinformatic tools helps improve our understanding of the regulatory function of *ABCF* in plant growth and development. Currently, the ABC gene family has been identified and analysed in several plants, including rice [5], flax [24], maize [36], pineapple [37], barley [38], capsicum [39], soybean [40], and other species [41,42,43]. However, to date, members of the ABCF gene family in wheat have not been systematically studied. Therefore, in the present study, we performed a basic genome-wide analysis of the *ABCF* family in wheat and investigated the mechanism of interaction between *TaABCF*s and plant viruses. We identified 18 *ABCF*s in wheat that were classified into four subfamilies, *TaABCF1*, *TaABCF2*, *TaABCF3*, and *TaABCF4*, by analysing their structural domains and referring to the classification of the *ABCF* family in *Arabidopsis thaliana* [23] (Figure 1). Seven *ZmABCFs* in *Z. mays* and six *OsABCFs* in *O. sativa* were identified by BLASTP. It can be seen that the *T. aestivum* genome had the highest number of ABCF genes amongst these species, with about three times as many genes as other diploid plant species. This may be due to its large genome size (about 17 Gb) and the allohexaploid (AABBDD) nature of its genome [44]. In addition, a relatively high number of ABCC genes has been reported in the tetraploid *Gossypium hirsutum* [45]. These results indicate that the emergence of a large number of ABCF genes may be attributed to their complex polyploid genome. Furthermore, a positive correlation between the number of genes and the ploidy level of the genome was observed. Phylogenetic analysis showed that ABCF proteins could also be classified into four subfamilies. The tight clustering of ABCF genes from *Arabidopsis*, *O. sativa*, *T. aestivum*, and *Z. mays* in all clades suggests that they evolved before the division of dicotyledonous and monocotyledonous plants. Moreover, the close clustering of homologous *TaABCF*s indicates their conservation, which was in accordance with purifying selection (Figure 2). After predicting protein structures, we found significant differences in the structures of ABCF1, ABCF2, ABCF3, and ABCF4 in wheat. We speculated that this might be due to the diversity in gene length. However, ABCFs exhibit structural conservation between wheat and *Arabidopsis*, indicating that ABCFs are highly homologous between the two species (Figure 3). After analysing the conserved motifs and gene structures, we identified 10 conserved motifs. The motif distribution patterns and gene structures of *TaABCF*s in the same subgroups were similar, but there were also some differences, indicating that genes in the same subfamily may have the same structures, but their functions are diverse (Figure 4).

Notably, gene duplication events play a vital role in the evolution as well as the expansion of gene families. They help organisms adapt to various environments and increase functional diversity. There are three types of gene duplications: tandem, segmental, and genome-wide duplications [46,47,48,49]. *TaABCF* has undergone duplication events during its evolution, which may confer a selective advantage under certain conditions. The results of the homology analysis showed that a total of 11 co-lineage events were identified in *TaABCF* (Figure 5). To investigate whether there was selective pressure acting on the *TaABCF*s and examine the types of selection pressures that affect duplicate gene pairs during evolution, we calculated the Ka, Ks, and Ka/Ks values for each homologous gene [29]. Ka/Ks values lower than 1 indicate a negative selection (purification), and Ka/Ks values over 1 indicate the presence of positive selection pressure [50]. Our results showed that the Ka/Ks values of the homologous duplicate gene pairs were far less than 1, suggesting that the *TaABCF* pairs were subjected to purification selection. In addition, we also estimated the divergence time. The Ks values indicated that replication events occurred from 0.0365 to 0.1111 Mya, which suggested that the duplication events occurred before the hybridisation events of the A, B, and D subgenomes. Analysis based on duplication events indicated that segmental duplication was the major factor leading to the amplification of the *ABCF* gene family. Cis-acting elements are involved in the regulation of gene transcriptional activity in plants and play important roles in plant hormone production, light-responsive activities, and developmental processes [51]. Therefore, we predicted cis-acting elements to explore the potential biological functions of *TaABCF* (Figure 6). The results showed that the promoter of *TaABCF* contains numerous cis-acting elements related to phytohormone response. For example, the ABRE, CGTCA-motif, TGACG-motif, GARE-motif, P-box, TCA-element, and TGA-element cis-acting elements are associated with plant hormone responses [52]. Amongst these, ABRE is an ABA response element, CGTCA-box and TGACG-motif are MeJA response elements, GARE-motif and P-box are GA response elements, TCA-element is an SA response element, and TGA-element is a cis-acting element related to IAA response. This suggests that *TaABCF* may be involved in multiple biotic and abiotic hormone-regulating processes. Our study also showed that the *TaABCF* promoter contained numerous cis-acting elements associated with biotic and abiotic stresses, such as ARE, G-box, GT1-motif, TCCC-motif, TCT-motif, LTR, and MBS. AREs are involved in anaerobically induced correlated responses [53], whereas MBS is an MYB-binding site involved in drought-response induction [54].

In addition, we revealed the tissue-specific expression patterns of *TaABCF*s (Figure 7). *TaABCF1* is highly expressed in the roots; therefore, we hypothesised that it may be involved in the development of root meristematic tissues. *TaABCF2* is highly expressed in the leaves, suggesting its role in leaf dynamics. *TaABCF3* and *TaABCF4* are stably expressed in various tissues, suggesting that these proteins are involved in multiple physiological processes in wheat. Previous studies have shown that ABC transporters play important roles in plant growth and development [55,56]. Therefore, *TaABCF* may be involved in the growth and development of wheat. The relative expression of *TaABCF* also changed under temperature stress, as our results showed that low temperatures suppressed its expression (Figure 8a). Thus, we speculate that the change in expression could promote the adaptation of wheat to the environment. Vernalisation is the process by which plant flowering is triggered after a sustained period of low temperature [57]. Studying the relationship between vernalisation and *TaABCF* may provide new directions for improving wheat yield.

Few studies have reported on the role of *TaABCF* in the response to plant viruses. CWMV is a major pathogen that causes typical mosaic symptoms in wheat. CWMV belongs to the family Virgaviridae and genus *Furovirus* [58] and is transmitted in nature by *Polymyxa graminis*, an obligate parasite in the roots of Gramineae plants [59]. Previous studies have indicated that the CWMV genome consists of two single-stranded positive-sense RNAs (RNA1 and RNA2). A full-length cDNA clone of CWMV has been constructed that can infect wheat and *Nicotiana benthamiana* [60]. Another major pathogen that causes wheat soil-borne mosaic disease is WYMV, which belongs to the genus *Bymovirus* (Potyviridae). The WYMV genome contains two positive single RNA strands: RNA1 (7.5 kb) and RNA2 (3.6 kb) [61]. Wheat infected with WYMV shows mosaic or yellow-striped leaves and plant stunting, and the grain yield is reduced by 20–70% in severely affected fields. BSMV is a single-stranded positive-sense RNA virus with a tripartite genome consisting of α, β, and γ RNAs [62]. BSMV is a vector of choice for silencing both host and pathogen genes, as well as the expression of the GOI (gene of interest) in various plant tissues, owing to its easy mechanical transmission and transmission via seeds [63]. Therefore, we investigated the changes in *TaABCF* expression after inoculation with BSMV, CWMV, and WYMV (Figure 9) to find more innovative and effective solutions to deal with CWMV, BSMV, and WYMV infections in wheat, which is the most crucial and widely used cereal grain crop. Previously, it was reported that the optimal temperature for CWMV infection is 17 °C [58], and the recessive symptom of WYMV and CWMV infection is the greening of leaves at approximately 24 °C [64]. The results showed that the expression of *TaABCF2* and *TaABCF4* was downregulated after WYMV infection. In contrast, *TaABCF2* was significantly upregulated after CWMV infection. Further verification of the relationship between *TaABCF* and different viruses is required to lay a foundation for future studies. The phytohormones ABA, SA, MeJA, and ethylene play major roles in mediating plant defence responses to pathogens and abiotic stresses [65,66]. Previous studies have shown that the SA-inducing peptides Zip1 [67] and *Arabidopsis* rapid alkalinisation factors [68] regulate immune response processes in maize. ABA can cause stomatal closure to maintain water homeostasis in plants under conditions such as high salinity and drought [69] and can promote long-term growth responses by regulating stress-response genes. In the present study, all *TaABCF*s were highly sensitive to MeJA; however, only *TaABCF1* and *TaABCF2* were sensitive to ABA (Figure 8b,c). Thus, we speculate that *TaABCF*s may participate in the immune response in wheat via the MeJA pathway; however, this requires further research. We found that silencing *TaABCF2* significantly reduced the resistance of wheat to CWMV infection, and the accumulation of CWMV RNA was notably increased in wheat leaves after silencing *TaABCF2* (Figure 10a–c). The accumulation of WYMV RNA also significantly increased, and the phenotypes were consistent with this result (Figure 10d–f). These results indicated that *TaABCF2* plays an essential role in the antiviral response of wheat. To further explore how *TaABCF2* plays a role in wheat disease resistance, we validated the interactions between *TaABCF2* and the proteins related to CWMV and WYMV. However, no related proteins were detected (Figure 10g,h). In this regard, we speculate that *TaABCF2* may not participate in the process of viral infection through direct interactions; however, further studies are required to investigate this mechanism.

In summary, we performed a genome-wide identification and functional analysis of the *TaABCF* gene family in wheat. CWMV and WYMV are the two major pathogens that cause soil-borne viral diseases in wheat, leading to a decrease in the yield and quality of wheat crops. Currently, the primary control method involves the cultivation of disease-resistant wheat varieties. This study is the first to report the molecular function of *TaABCF2* during CWMV and WYMV infections, explore the molecular mechanisms involved in CWMV and WYMV infections, and provide a direction for the identification of soil-borne viral resistance genes in wheat. To better comprehend the function of *TaABCF2*, we intend to investigate the mechanism by which *TaABCF2* responds to viruses in plants in the future.

## 4. Materials and Methods

### 4.1. Genome-Wide Identification of TaABCF Family Genes

The protein sequences of *Arabidopsis* ABCF family members (*At1g60790*, *At5g09930*, *At1g64550*, *At3g54540*, and *At5g64840*) were downloaded from the National Center for Biotechnology Information (NCBI) database (https://www.ncbi.nlm.nih.gov, accessed on 3 April 2023) and used as BLASTP templates to identify all ABCF proteins in wheat, rice, and maize. Genomic data for wheat, rice, and maize were obtained from the Ensembl Plants Database (https://plants.ensembl.org/Triticum_aestivum/Info/Index, accessed on 3 April 2023). A total of 18 wheat homologues with Eval < 10^−6^ and %ID > 60 were screened, along with 6 rice homologues and 7 maize homologues meeting the same criteria. All candidate proteins were further analysed using the Pfam database (https://pfam.xfam.org/, accessed on 3 April 2023) [70] and the NCBI Batch Web CD-Search Tool (https://www.ncbi.nlm.nih.gov/Structure/bwrpsb/bwrpsb.cgi, accessed on 3 April 2023) [71]. Detailed information on the *TaABCF*s, including the amino acid counts, chromosome positioning, and coding sequence lengths, was obtained from the Ensembl Plants Database. The molecular weight and theoretical isoelectric point of each TaABCF protein were obtained using ExPAsy (https://web.expasy.org/compute_pi/, accessed on 3 April 2023).

### 4.2. Multiple Sequence Alignment and Phylogenetic Tree Construction

Phylogenetic analyses were performed using sequences from the Ensembl Plants Database and NCBI database. The identified TaABCF, OsABCF, and ZmABCF protein sequences and five AtABCF protein sequences downloaded from NCBI were imported into MEGA-X software (Version: 10.0) [72] for multiple sequence alignment using the MUCLE function and then used to construct an unrooted phylogenetic tree with the neighbour-joining method and 1000 bootstrap replicates. The online tool EvolView (https://evolgenius.info/evolview-v2/#login, accessed on 5 April 2023) was used to create improved graphical representations of trees.

### 4.3. Structural Prediction of TaABCF Protein

We predicted the structures of the TaABCF proteins using the SWISS-MODEL (https://swissmodel.expasy.org/, accessed on 5 April 2023) homology model [25]. One gene was randomly selected from each group and species for protein modelling as follows: *ABCF1* (*AT1G60790*, *TraesCS7B02G072300.1*); *ABCF2* (*AT5G64840*, *TraesCS4A02G156300.1*); *ABCF3* (*AT1G64550, TraesCS6A02G409800.1*); and *ABCF4* (*AT3G54540*, *TraesCS3B02G388600.1*).

### 4.4. Gene Structural Domain, Gene Structure, and Motif Analysis of TaABCF

The TaABCF protein sequence was submitted to the NCBI Batch CD-Search tool (https://www.ncbi.nlm.nih.gov/Structure/bwrpsb/bwrpsb.cgi, accessed on 5 April 2023), and the gene structure domain data were obtained and visualised using TBtools. Gene annotation files for wheat were downloaded from the Ensembl Plants Database (http://plants.ensembl.org/index.html, accessed on 5 April 2023) [73], and gene structure was analysed using TBtools Gene Structure View (Version: 1.098768, Advanced). The motifs of *TaABCF* were predicted using the MEME Suite 5.2.3 (https://meme-suite.org/meme/tools/meme, accessed on 5 April 2023) online analysis website [74]. Ten conserved motifs were also identified.

### 4.5. Chromosome Localisation and Gene Duplication

Information on the chromosomal loci of *TaABCF* was retrieved from the Ensembl Plants Database (http://plants.ensembl.org/Triticum_aestivum/Location/Genome, accessed on 7 April 2023). The wheat genome annotation file was downloaded (ftp://ftp.ensemblgenomes.org/pub/release-47/plants/gtf/triticum_aestivum, accessed on 7 April 2023), and TBtools MCScanX (Version: 1.098768) was used to analyse the chromosomal localisation of *TaABCF* and the gene duplication events in the wheat genome [75].

### 4.6. Calculation of Ka/Ks Values

The Ka/Ks value indicates the ratio between the non-synonymous (Ka) and synonymous (Ks) substitution rates of two protein-coding genes. The Ka/Ks ratio of the homologous gene pairs was used to determine whether they were under selection. When Ka/Ks was > 1, the gene obeyed positive selection; Ka/Ks = 1 indicated neutral evolution, and Ka/Ks < 1 indicated purifying selection. The Ka/Ks ratio was calculated using TBtools software, and the scatter time (T) [29] was calculated based on T = Ks/(2 × 9.1 × 10^−9^)Mya.

### 4.7. Prediction of Cis-Acting Elements in the Promoter Region

The promoter sequences containing the 2000 bp region upstream of all *TaABCF* genes were downloaded from the Ensembl Plants Database. The promoter regions were identified in cis-acting regulatory elements using PlantCARE software (http://bioinformatics.psb.ugent.be/webtools/PlantCARE/html/, accessed on 8 April 2023) [76].

### 4.8. Plant Material, Growth Conditions, and Virus Inoculation

The materials used in this study, including Yangmai 158 (a virus-resistant wheat variety) and *Nicotiana benthamiana*, were provided by Dr. Jian Yang. Yangmai 158 and *Nicotiana benthamiana* plants were grown in an artificial greenhouse at 25 °C with a 16 h light/8 h dark cycle, and the wheat was subjected to stress treatments when it grew to the three-leaf stage. In the temperature stress treatments, the plants were placed in growth cabinets at different temperatures (8, 15, 20, and 25 °C) with a 16 h light/8 h dark cycle, and wheat at 8 °C was used as a control. For the virus inoculation treatments, BSMV, CWMV, and WYMV were inoculated via in vitro transcription and mechanical rubbing. A BSMV-based gene vector was provided by Dr. Dawei Li (Shanghai, China) [77]. CWMV- and WYMV-based gene vectors were provided by Dr. Jian Yang [61]. The three viruses had the same inoculation method. In the case of BSMV, firstly, the plasmid transcripts of BSMV RNA α, β, and γ were linearised for in vitro transcription. Secondly, the linearised plasmids were mixed with equal amounts of excess inoculation buffer (FES: 0.06 M potassium phosphate, 0.1 M glycine, 1% bentonite, 1% sodium pyrophosphate decahydrate, 1% feldspar, pH 8.5) in a molar ratio of 1:1:1 [61,77]. Finally, the mixture was inoculated onto the leaves of the wheat seedlings at the three-leaf stage by mechanical rubbing. Plants inoculated with FES alone (MOCK) were used as negative controls. In the case of WYMV, three biological replicates of the two wheat groups (wheat inoculated with WYMV and untreated wheat) cultured under the same conditions were used for the gene expression analysis. Untreated wheat plants were used as negative controls. Fourteen days later, after confirming successful infection with WYMV, wheat leaves were collected for RT-qPCR analysis, and four *TaABCF* genes were randomly selected to assess changes in their expression after virus infection. GraphPad Prism 9.5 software was used to display the analysis results. CWMV was inoculated in the same manner as WYMV.

### 4.9. Plant RNA Isolation and RT-qPCR Assay

RNA was extracted from each sample using the HiPure Plant RNA Mini Kit (Magen, Guangzhou, China) and stored at −80 °C until direct use. First-strand cDNA was synthesised using the First Strand cDNA Synthesis Kit (Toyobo, Kita-ku, Osaka, Japan) by adding 1 μg of total RNA per 20 μL of reaction system [78]. RT-qPCR analysis was performed using an ABI 7900HT Sequence Detection System (Applied Biosystems, Foster City, CA, USA) and Hieff qPCR SYBR Green Master Mix (Yeasen, Shanghai, China). The RT-qPCR program was 5 min at 95 °C, followed by 40 cycles of 15 s at 95 °C, 20 s at 62 °C, and 30 s at 72 °C. The 2^−ΔΔCt^ method [79] was used to examine the relative expression of *TaABCF*, and the *T. aestivum* cell division cycle (TaCDC) gene (accession number: XM_020313450) was used as an internal reference gene [61]. At least three biological and three technical replicates were used for each treatment. Primers were designed using NCBI Primer-BLAST (http://www.ncbi.nlm.nih.gov/tools/primer-blast, accessed on 8 May 2023). The list of primers used is provided in Supplementary Table S2. Data were visualised as histograms using GraphPad Prism 9.5 software.

### 4.10. Tissue-Specific Expression of TaABCF

Four *TaABCF* genes were randomly selected, and their expression was analysed in five different wheat tissues. Wheat plants were divided into five tissue types: the top leaves, middle leaves, bottom leaves, stems, and roots. Three replicates of each wheat tissue sample were collected and stored at −80 °C until total RNA was extracted. Gene expression was determined using RT-qPCR. Briefly, qRT-qPCR was performed as follows: one cycle at 95 °C for 5 min; followed by 37 cycles at 95 °C for 30 s, 58 °C for 30 s, and 72 °C for 1 min; and then one cycle at 72 °C for 8 min, with single-point fluorescence detection at 72 °C. The results of the tissue-specific expression analysis were displayed using GraphPad Prism 9.5.

### 4.11. Expression of TaABCF under ABA and MeJA Treatments

Wheat plants at the three-leaf stage (Yangmai 158) were treated with 100 μmol L^−1^ abscisic acid (ABA) and 100 μmol L^−1^ MeJA as exogenous sprays, as described in Yu et al. [80]. Wheat treated with distilled water was used as a control. Three biological replicates of the samples were collected at four different time points (2, 4, 6, and 12 h), and the samples were stored at −80 °C after collection until total RNA was extracted. The expression of each gene was determined using RT-qPCR. The results were analysed using GraphPad Prism 9.5.

### 4.12. Virus-Induced Gene Silencing (VIGS) in Wheat

The best VIGS fragment sequence (300 bp) of *TaABCF2* was obtained using the VIGS tool function online (https://solgenomics.net/, accessed on 10 April 2023). The fragment was inserted into the pBSMVγ vector. Plasmids pBSMVα, pBSMVβ, pBSMVγ, pBSMVγ:TaABCF2, and pBSMVγ:TaPDS were linearised separately using specific restriction endonucleases. The transcription products were mixed at a molar ratio of 1:1:1 using the T7 in vitro Transcription Kit (Ambion, Austin, TX, USA), and then FES buffer was added; each second leaf of two-leaf-stage wheat plants was inoculated with 10 μL of the mixed transcripts by friction [81]. Wheat plants inoculated with FES buffer were used as negative controls, and those inoculated with the phytoene desaturase gene exhibiting a typical photobleaching phenotype were used as positive controls. The inoculated wheat seedlings were grown under dark conditions at 28 °C and 70% relative humidity for 24 h. After 7 d of growth under a 16 h light/8 h dark photoperiod, they were inoculated again with CWMV or WYMV and grown under greenhouse conditions at 15 °C and 70% relative humidity for 14 d. RNA was extracted from the virus-infected leaves at 7 dpi. Photographs of the virus-infected leaves were taken at 21 dpi.

### 4.13. Yeast Two-Hybrid (Y2H) Assay

The coding sequences of wheat *TaABCF2* (accession number: TraesCS4B02G160600.1) were cloned and inserted into the Gal4-activating domain (vector: pGADT7) named pAD-TaABCF2. The coding sequences of seven proteins from CWMV RNA (MP, P153, Rd4, CRP, CP, NCP, and CPRT) and 10 proteins from WYMV RNA (P3, CI, VPg, NIa, NIb, 7K, 14K, CP, P1, and P2) were cloned; fused to the Gal DNA-binding domain (vector: pGBKT7); and named pBD-CRP, pBD-MP, pBD-CP, pBD-P153, pBD-Rd4, pBD-NCP, pBD-CPRT, pBD-NIa, pBD-NIb, pBD-7K, pBD-14K, pBD-P1, pBD-P2, pBD-P3, pBD-CI, pBD-CP, and pBD-VPg. The pAD-TaABCF2 plasmids with each pBD-recombinant plasmid were co-transformed into yeast strain Y2H Gold following the TaKaRa protocol handbook (TaKaRa Bio Inc, Japan). The transformed yeast was plated onto a low-stringency selective medium lacking tryptophan and leucine (SD/-Leu/-Trp), incubated for 72 h, and then transferred onto a high-stringency selective medium lacking tryptophan, leucine, histidine, and adenine (SD/-Trp/-Leu/-His/-Ade) for 3–5 d. Yeast cells co-transformed with AD-T and BD-lam were used as negative controls, whereas yeast cells co-transformed with AD-T and BD-53 were used as positive controls.

## 5. Conclusions

In this study, we identified 18 members of the *ABCF* family in wheat and demonstrated that they can be categorised into four groups (*ABCF1*, *ABCF2*, *ABCF3*, and *ABCF4*). Members of the same subfamily may have similar functions based on their gene structures and conserved domains. We performed a comprehensive genome-wide analysis of the protein patterns, chromosomal positions, introns, and exons of *TaABCF* family members. In addition, we analysed the expression profiles of *TaABCF*s under different temperatures, hormones, and plant viral stresses. The results showed that *TaABCF2* was significantly upregulated after inoculation with CWMV but significantly downregulated after inoculation with WYMV. After silencing *TaABCF2*, the accumulation of both CWMV and WYMV RNA in wheat leaves increased significantly, indicating that *TaABCF2* is involved in the wheat antiviral response and laying the foundation for subsequent research.

## Figures and Tables

**Table 1:** Detailed information about 18 predicted ABCF proteins in *Triticum aestivum*.

Gene ID	Location	CDS Length (bp)	Size (aa)	MW (kDa)	PI	Exons	Group
TraesCS7B02G072300.1	7B: 79,386,663–79,390,314	2544	1027	106.42	8.48	5	ABCF1
TraesCS7A02G167500.1	7A: 122,759,312–122,762,749	2550	849	88.69	6.29	5	ABCF1
TraesCS7D02G168300.1	7D: 118,824,218–118,828,023	2553	850	88.59	6.67	5	ABCF1
TraesCS2B02G381000.1	2B: 545,154,783–545,157,775	2364	787	85.31	9.45	5	ABCF1
TraesCS2A02G363100.1	2A: 607,514,687–607,517,465	2346	781	85.01	9.28	5	ABCF1
TraesCS2D02G360400.1	2D: 462,242,110–462,244,920	2391	796	86.55	9.41	5	ABCF1
TraesCS4A02G156300.1	4A: 311,618,303–311,664,820	2079	692	77.69	7.09	9	ABCF2
TraesCS4D02G149400.1	4D: 155,574,671–155,578,885	2079	692	77.57	7.9	9	ABCF2
TraesCS4B02G160600.1	4B: 315,061,175–315,067,581	2079	692	77.53	6.78	9	ABCF2
TraesCSU02G090500.1	Un: 81,296,469–81,302,814	2163	720	80.13	6	15	ABCF3
TraesCS6A02G409800.1	6A: 613,481,932–613,488,341	2163	720	80.14	5.95	16	ABCF3
TraesCS6B02G456000.1	6B: 712,345,436–712,351,600	2163	720	80.18	6.08	14	ABCF3
TraesCS1D02G409400.1	1D: 471,300,960–471,303,080	2121	706	77.33	6.51	1	ABCF4
TraesCS1B02G430800.1	1B: 655,102,218–655,104,350	2133	710	77.61	6.5	1	ABCF4
TraesCS7D02G054900.1	7D: 29,535,278–29,537,442	1677	558	61.55	6.13	4	ABCF4
TraesCS3A02G355700.1	3A: 603,789,869–603,792,554	2169	722	78.63	6.51	1	ABCF4
TraesCS3B02G388600.1	3B: 611,676,317–611,678,940	2169	722	78.71	6.5	1	ABCF4
TraesCS3D02G349700.1	3D: 461,199,491–461,201,659	2169	722	78.69	6.4	1	ABCF4

CDS, coding sequence; bp, base pair; aa, amino acid; MW, molecular weight; Da, Dalton; PI, isoelectric point.

**Table 2:** Ks, Ka, and Ka/Ks values calculated for paralogous *ABCF* gene pairs (*T. aestivum–T. aestivum*).

Paralogous		Ka	Ks	Ka/Ks	T (Mya)
TraesCS1B02G430800.1	TraesCS1D02G409400.1	0.0056	0.0756	0.0739	4.153
TraesCS2A02G363100.1	TraesCS2B02G381000.1	0.0385	0.0692	0.5566	3.801
TraesCS2A02G363100.1	TraesCS2D02G360400.1	0.0345	0.0947	0.3646	5.201
TraesCS2B02G381000.1	TraesCS2D02G360400.1	0.0353	0.0805	0.4385	4.424
TraesCS3A02G355700.1	TraesCS3B02G388600.1	0.0036	0.1111	0.0327	6.107
TraesCS3A02G355700.1	TraesCS3D02G349700.1	0.0036	0.0737	0.0494	4.050
TraesCS3B02G388600.1	TraesCS3D02G349700.1	0.0049	0.0910	0.0534	4.998
TraesCS4A02G156300.1	TraesCS4B02G160600.1	0.0110	0.0444	0.2482	2.438
TraesCS4B02G160600.1	TraesCS4D02G149400.1	0.0082	0.0365	0.2238	2.007
TraesCS6A02G409800.1	TraesCS6B02G456000.1	0.0036	0.0667	0.0543	3.666
TraesCS7A02G167500.1	TraesCS7B02G072300.1	0.0345	0.1057	0.3265	5.809
TraesCS7A02G167500.1	TraesCS7B02G072300.2	0.0327	0.0961	0.3403	5.281
TraesCS7B02G072300.1	TraesCS7B02G072300.3	0.0318	0.1078	0.2951	5.920

## Data Availability

Data are contained within the article and Supplementary Materials.

## Supplementary Materials

The following supporting information can be downloaded at: https://www.mdpi.com/article/10.3390/ijms242216478/s1.
