# Supplementary material for: Genome-Wide Identification and Analysis of the ABCF Gene Family in Triticum aestivum
Source: Int J Mol Sci. 2023 Nov 18;24(22):16478. doi: 10.3390/ijms242216478 (PMC10671407; doi:10.3390/ijms242216478)
Supplement: Supplementary file 1 [file ijms-24-16478-s001.zip › Supplementary Figures.pdf]

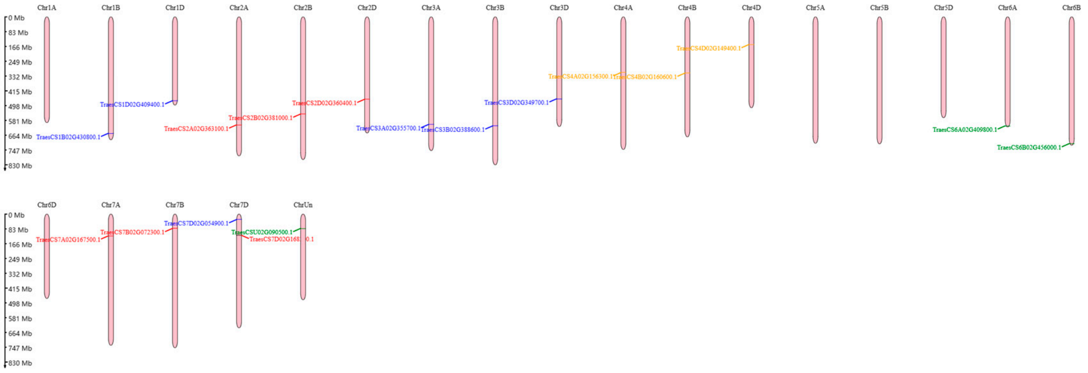

Figure S1. Chromosome locations of *TaABCF* genes. Chromosomes are represented by cylinders. The red font represents the *ABCF1* group, the orange font represents the *ABCF2* group, the green font represents the *ABCF3* group, the blue font represents the *ABCF4* group.

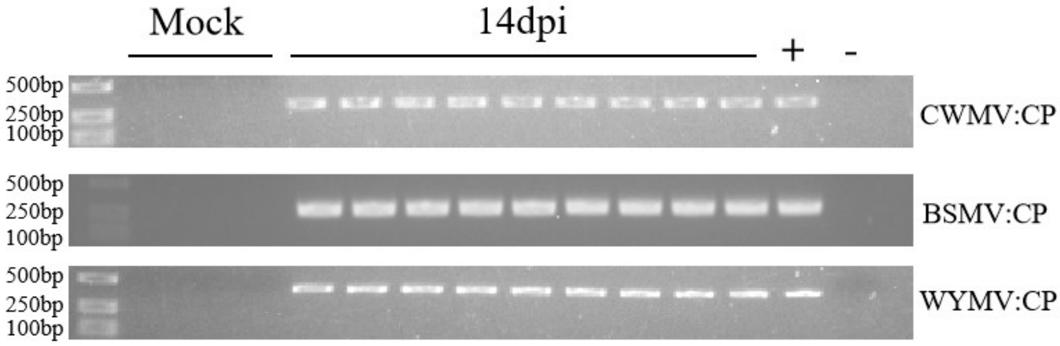

Figure S2. Reverse transcription PCR (RT-PCR) detection of BSMV, CWMV and WYMV infections. Three plants were analyzed for each treatment. Total RNA from health wheat plant was used as a negative control (−). Diluted plasmid BSMV-β, CWMV RNA 2, WYMV RNA 1 were used as the positive control (+) for BSMV, CWMV and WYMV, respectively.

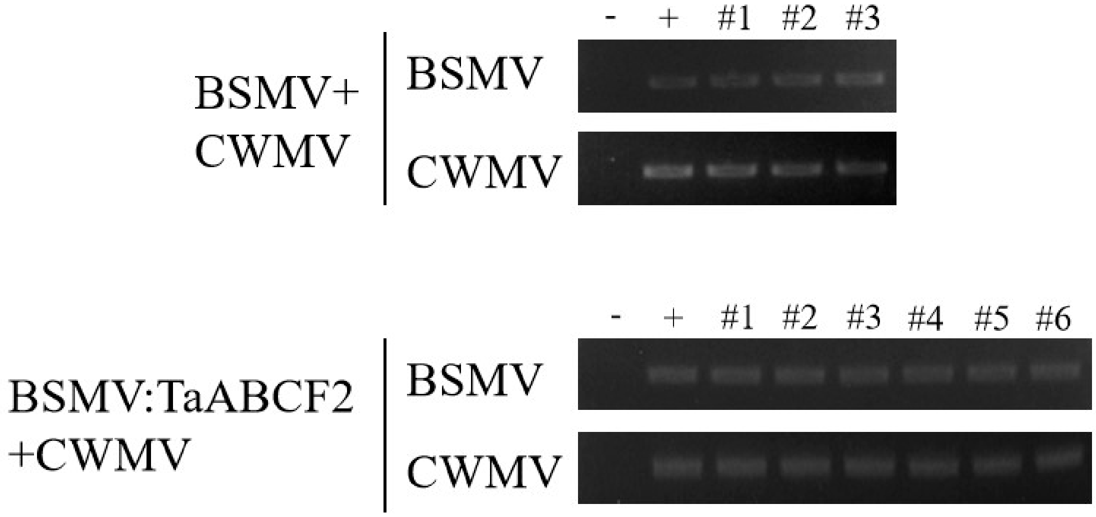

Figure S3. Detection of CWMV and BSMV by RT-PCR in virus infected leaves. The infectious clones and wheat leaves without viral infection was used as positive control (+) and negative control (-), respectively.

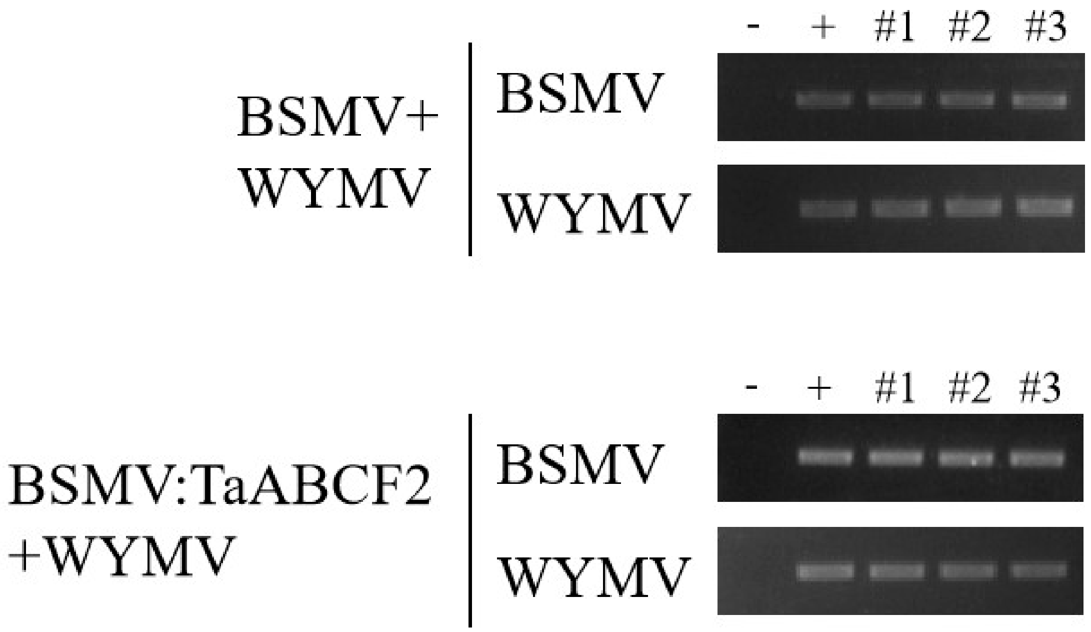

Figure S4. Detection of WYMV and BSMV by RT-PCR in virus infected leaves. The infectious clones and wheat leaves without viral infection was used as positive control (+) and negative control (-), respectively.
